# Factors associated with hospital admission and 30-day readmission for children less than 18 years of age in 2018 in France: a one-year nationwide observational study

**DOI:** 10.1186/s12913-023-09861-2

**Published:** 2023-08-23

**Authors:** Jeanne Pergeline, Sylvie Rey, Jeanne Fresson, Gonzague Debeugny, Antoine Rachas, Philippe Tuppin

**Affiliations:** 1 Caisse Nationale de l’Assurance Maladie, Direction de la Stratégie des Etudes et des Statistiques, F-75986 Paris Cedex 20, France; 2 Direction de la Recherche, des Etudes, de l’Evaluation et des Statistiques (Drees), 75015 Paris, France

**Keywords:** Children, Short stay hospital, Healthcare services use, Conditions, Sociodeprivation

## Abstract

**Background:**

Nationwide data for children for short-stay hospitalisation (SSH) and associated factors are scarce. This retrospective study of children in France < 18 years of age followed after their birth or birthday in 2018 focused on at least one annual SSH, stay < 1 night or ≥ 1 night, or 30-day readmission ≥ 1 night.

**Methods:**

Children were selected from the national health data system (SNDS), which includes data on long-term chronic disease (LTD) status with full reimbursement and complementary universal coverage based on low household income (CMUC). Uni and multivariate quasi-Poisson regression were applied for each outcome.

**Results:**

Among 13.211 million children (94.4% population, 51.2% boys), CMUC was identified for 17.5% and at least one LTD for 4% (0-<1 year: 1.5%; 14-<18 year: 5.2%). The most frequent LTDs were pervasive developmental diseases (0.53%), asthma (0.24%), epilepsy (0.17%), and type 1 diabetes (0.15%). At least one SSH was found for 8.8%: SSH < 1 night (4.9%), SSH ≥ 1 night (4.5%), readmission (0.4%). Children with at least one SSH were younger (median 6 vs. 9 years) and more often had CMUC (21%), a LTD (12%), an emergency department (ED) visit (56%), or various primary healthcare visits than all children. Those with a SSH ≥1 night vs. < 1 night were older (median: 9 vs. 4 years). They had the same frequency of LTD (13.4%) but more often an ED visit (78% vs. 42%). Children with readmissions were younger (median 3 years). They had the highest levels of CMUC (29.3%), LTD (34%), EDs in their municipality (35% vs. 29% for the whole population) and ED visits (87%). In adjusted analysis, each outcome was significantly less frequent among girls than boys and more frequent for children with CMUC. LTDs with the largest association with SSH < 1 night were cystic fibrosis, sickle cell diseases (SCD), diabetes type 1, those with SSH ≥1 night type 1 diabetes epilepsy and SCD, and those for readmissions lymphoid leukaemia, malignant neoplasm of the brain, and SCD. Among all SSH admissions of children < 10 years, 25.8% were potentially preventable.

**Conclusion:**

Higher SSH and readmission rates were found for children with certain LTD living in low-income households, suggesting the need or increase of specific policy actions and research.

**Supplementary Information:**

The online version contains supplementary material available at 10.1186/s12913-023-09861-2.

## Background

The utilisation of healthcare services by children varies between and within countries and depends on multiple factors, including household socioeconomic deprivation and neighbourhood inequalities and the level of healthcare coverage, healthcare supply, and organisation, in particular that devoted to children, and, of course, health status and disease epidemiology and their consequences on child development [1–3]. As for adults, large sample studies on children have mostly focused on the evolution of utilisation rates, the characteristics associated with an increased risk of hospital admission or readmission, high users of hospital services, costs, and quality indicators. However, definitions have varied between studies. Changes and evolution in healthcare to achieve its more rational use must be designed to limit potentially preventable conditions that lead to hospitalisation or ambulatory care thought to be avoidable if timely and adequate non-hospital care is provided in settings with universal healthcare. Furthermore it will be necessary to further develop ambulatory and one-day care and limit 30-day readmissions to shift healthcare away from SSH to community and ambulatory services. Studies on hospital admissions and readmissions have focused mainly on those that were unplanned as a quality indicator, as well as extra-costs and evaluations of the use of healthcare coverage [4–10].

Readmission rates are used as a quality indicator, and vary between hospitals [11, 12]. Risk factors associated with hospital admissions and readmissions have been described and many are common, such as being a young infant or adolescent, comorbidity, and public health insurance coverage [13–15]. Moreover, several studies have also focused on the hospitalisation of children with complex or chronic conditions and their healthcare use, but the reported prevalence varies depending on the definitions and criteria used [12, 16–18]. Increasing one-day admissions and ambulatory care and decreasing avoidable readmissions and admissions and the length of stay are means to decrease hospital costs. Thus, indicators on the large population must be followed to survey the occurrence of adverse effects of actions taken to optimize or limit these types of stays.

Nevertheless, in France, quasi-exhaustive and detailed nationwide estimations of short stay hospitalisations (SSHs), detailing one-day stays and readmission rates for children according to their health condition and sociodemographic characteristics are lacking. The French national health data system (SNDS: système national des données de santé) can be used for such studies because it includes data on individual characteristics, reimbursed primary care prescriptions, and hospitalisations for almost the entire population, which has universal health insurance coverage [19].

The primary objective of this first French nationwide observational study on children under the age of 18 years in 2018 was to describe the various types of SSHs, globally and by age, and the factors associated. The reported frequency of primary care use and hospital diagnoses detailing those that were potentially preventable are also reported for each SSH type.

## Methods

### Study design and population

This retrospective observational cohort included children < 18 years of age in 2018 and followed up for one year between 2018 and 2019 according to their birth or birthday. In mainland France (population of 64.9 million), there were 13.99 million children < 18 years of age on 1 January 2019, according to the National Institute for Statistics and Economic Studies (Institut national de la statistique et des études économiques) [20]. Those excluded from the analysis included children who did not receive any refund during the one-year follow-up period and those of the same sex resulting from a multiple birth (twins, triplets, etc.) who could not be unambiguously identified in the database, as well as those with a false number of linkages or those lacking critical information, such as the month of birth (N = 13.5 million remaining). For the sake of homogeneity, we also excluded children of different sexes resulting from multiple births (N = 13.219 million). We also excluded children whose month of birth could not be identified (N = 13.214 million) and those who died during the follow-up. Finally, 13.211 million children were included (94.4% of the total population estimated National Institute for Statistics and Economic Studies). In summary, this study concerned singleton children living in mainland France with national health insurance coverage who had at least one healthcare reimbursement from health insurance in 2018.

### Data source

The SNDS collects comprehensive anonymous data concerning all reimbursed prescriptions, examinations, and procedures performed in the outpatient setting and hospitalisations. Information about the beneficiaries themselves (date of birth, sex, place of residence, deprivation index, etc.) are also available [19]. This database does not record primary medical consultation diagnoses or the results of clinical examinations and investigations. Nevertheless, information is available concerning chronic long-term disease status (LTD). The LTD status is requested or renewed by the patient’s general practitioner (GP) and guarantees 100% reimbursement for all healthcare expenditures related to the LTD for at least five years. Diagnoses are made and validated after medical examination and, in certain cases, hospitalisation or chronicization during follow-up, by the patient’s GP using national recommendations for diagnosis, treatment, and follow-up. The list of LTDs is published and updated by decree after expertise from the Haute Autorité de Santé (French National Authority for Health) [21, 22]. A guide is also sent to the patient and information is also available on the French National Authority for Health website [22]. Through a pseudonymised identification number, this information is all linked via the national hospital discharge database to data concerning public and private hospital stays: emergency department (ED), SSH, psychiatric hospitals or rehabilitation facilities, home hospitalisation. Hospital and LTD diagnoses are coded according to the International Classification of Diseases 10th revision (ICD-10). Normal stays in hospitals/maternity units for childbirth without the need for neonatal care were excluded. The one-day services provided are equivalent in nature, complexity, and the medical supervision they require to services generally provided in the context of full-time hospitalisation. Healthcare institutions cannot claim hospitalization funding for services that can be provided in the form of outpatient care.

A social geographical deprivation index (FDep) has been created at the municipality scale (smallest administrative unit, 36,000 units in mainland France) according to four factors resulting from data published by the National Institute for Statistics and Economic Studies [23]: average household income, the percentage of secondary school graduates (those who finished secondary school in France who have passed the Baccalaureate exam) among inhabitants aged 15 and over, the percentage of blue-collar workers in the active population, and unemployment levels. It was computed in 2015 and is divided into five quintiles (1: most deprived, 5: least deprived). Information on the type of municipality of residence (urban or rural) is also present in the SNDS. Data concerning the presence of an emergency department (ED) in the municipality of residence on December 31, 2019, are available from the Ministry of Health (DREES open data).

An additional social deprivation marker is being the recipient, or not, of complementary universal health coverage (CMUC), which is granted for one year (renewable) based on annual income to those who have had a stable and regular residence in France for over three months [24]. The household may include the applicant, his/her spouse, partner, and children. In 2018, the annual income limit was 8,810 euros for a single person and it increased according to the number of people in the household. This limit is below the poverty threshold, defined as 50% of median income, or 10,620 euros in France in 2018. The CMUC enables beneficiaries to access treatment without advancing costs, with a reimbursement of 100% and without exceeding reimbursed costs. Children were classified as a CMUC beneficiary if they had at least one specific outpatient reimbursement covered by the CMUC in 2018 or 2019.

### Outcome measures

The outcomes were at least one SSH during the year of follow-up, one SSH < 1 night, one SSH ≥ 1 night, or one SSH readmission ≥ 1 night within 30 days of discharge (regardless the length of the initial stay).

### Statistical analysis

We conducted a descriptive analysis for each outcome group using information detailed above. Having an LTD status was considered for children with at least one of the most frequent LTDs for each outcome, detailed by diagnosis, or those with one or more of the less frequent LTDs, pooled as other LTD. The results for the use of primary or other healthcare services during the year are expressed by group as the rate of individuals who used such services at least once during the year of follow-up. The median number of instances of healthcare use is reported, together with the interquartile range (IQR), to assess the intensity of healthcare use during the year of follow-up. Data on the SSH principal diagnosis are expressed as the proportion of hospitalisations for the corresponding ICD-10 chapter among all hospitalisations and the most frequent specific conditions for certain chapters by group. Children with a SSH stay < 1 night and of another type were counted in each group. Data on potentially preventable admissions are expressed as the proportion of SSHs of the corresponding diagnosis among children between the age of 0 and 10 years based on a tool developed in New Zealand [25, 26].

Quasi-Poisson regression models were used to identify factors associated with the various outcomes of stay or readmission. Social deprivation was not considered in the full analysis due to collinearity with the variable CMUC and was only adjusted for age and sex. This was also true for the class “at least one LTD”, because the 10 most frequent LTD diagnoses were detailed in the full model analysis with the “other LTD diagnoses” class. The crude relative risk (RR) and 95% confidence interval (95%CI) are presented for each outcome in the supplementary material and then adjusted in the full models in the text. SAS (version 7.13, SAS Institute Inc, Cary, NC, USA) and R software (4.1.2) were used for the statistical analysis.

## Results

### Hospital admission rates

**Fig. 1:**
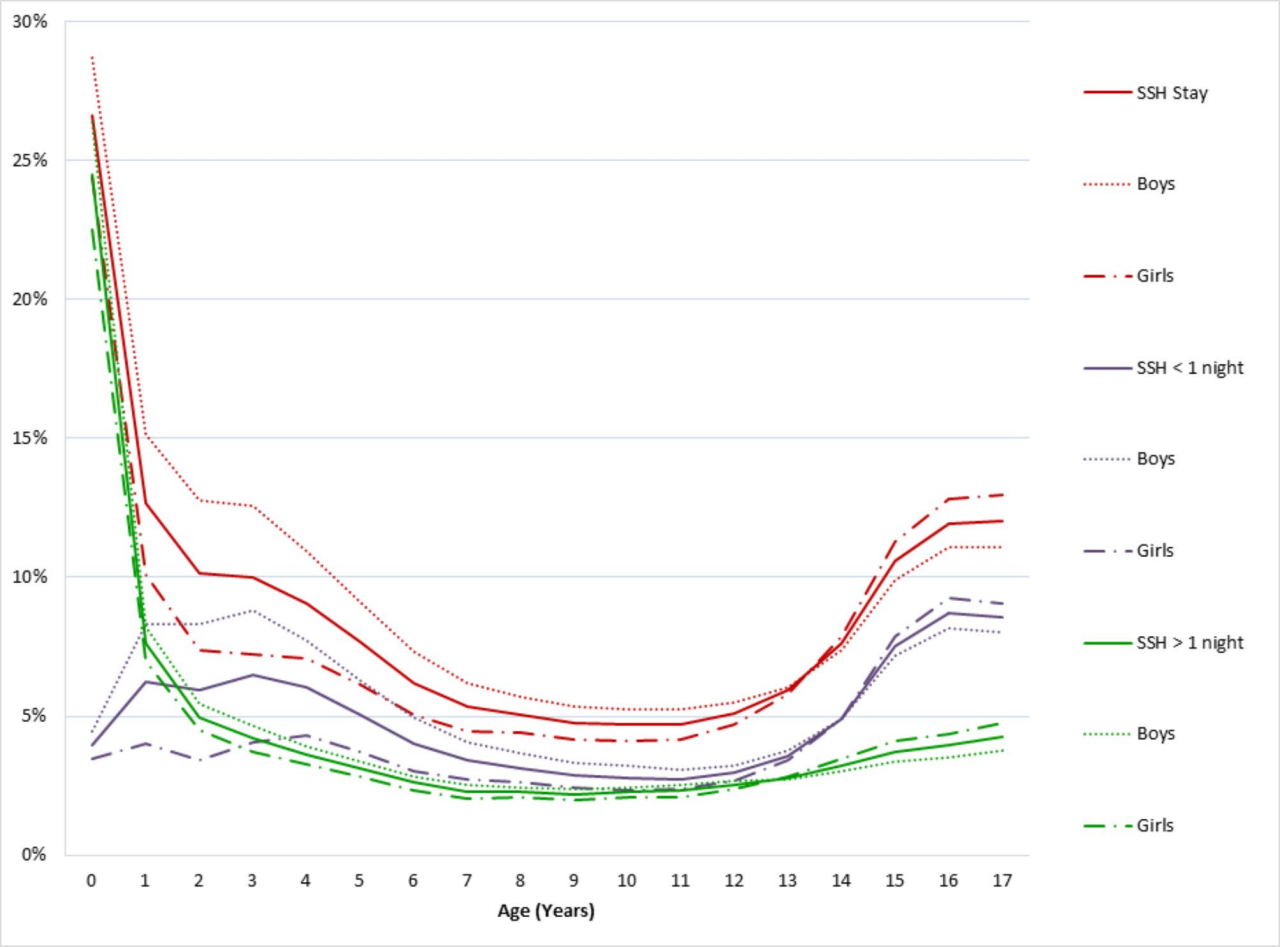
Rate of at least one hospitalisation of children < 18 years of age in 2018 and followed for one year after their birthday or birth, according to age, sex, and mode of hospitalisation. SSH: short stay hospitalisation (all, less than one night, or one night or more)

Our study included 13.211 million children (48.8% girls) under the age of 18 years (Median 9 years [IQR = 4–13]) living in mainland France in 2018. We found at least one SSH admission during the year, regardless of the length, for 8.8% of children, which was higher before one year of age (27%) and in adolescence (10.6% between 14 and 17 years) than between 10 and 13 years (5.1%) (Fig. 1). Similar proportions were found for SSH ≥ 1 night (4.4% of children): 24.5%, 3.8%, and 2.5%, respectively. There were two peaks for SSH < 1 night (4.9% of children): 6.2% between 2 and 4 years and 7.4% between 14 and 17 years. Boys more often had a SSH < 1 night before 12 years of age than girls, mainly between the ages of 1 and 4 years. At least one 30-day readmission ≥ 1 night was observed for 4.8% of children admitted at least once for a SSH. It was more frequent for the youngest children (0–1 year: 9.6%), following by adolescents (10–14 years: 4.5%).

### Child characteristics and LTDs

The median age of children admitted at least once for a SSH < 1 night was 9 years (Table 1). Those who had a SSH ≥ 1 night or readmitted ≥ 1 night were younger (median of 4 and 3 years, respectively). CMUC was identified for 17.5% of children, more frequently for those who were hospitalised or readmitted (24% and 29%). This was also found for the most deprived quintile of the deprivation index. Overall, 22% of children lived in a rural municipality (19% for 30-day readmission ≥ 1 night) and 29% in a municipality with an ED (35% for readmission).

**Table 1:** Characteristics and most frequent long-term disease diagnoses of children < 18 years of age who had at least one short stay hospitalisation, according to length or readmission

	At least one					
	Total	SSH stay	SSH stay < 1 night	SSH stay ≥ 1 night	SSH readmission ≥ 1 night	
**N**	13,211,235	1,157,701	650,043	588,631	55,509	Rate 4.8%
	%	%	%	%	%	
**Age** (Years)						
Median [IQR]	9 [4–13]	6 [2–14]	9 [4–15]	4 [0–12]	3 [0–12]	-
0 - < 1 year	5.1	15.5	4.1	28.1	31.1	9.6
1 - < 2	5.3	7.7	6.7	9.0	9.0	5.6
2 - < 5	16.4	18.2	20.5	15.6	13.8	3.6
5 - < 10	28.4	18.8	21.4	15.9	13.8	3.5
10 - < 14	22.7	13.2	13.9	12.6	12.4	4.5
14 - < 18	22.1	26.6	33.4	18.8	19.8	3.6
**Girls**	48.8	44.0	41.7	46.5	46.6	5.1
**Complementary universal health insurance coverage**	17.5	21.4	19.2	24.4	29.3	6.6
* Missing*	0.1	0.1	0.0	0.1	0.1	4.8
**Geographical social deprivation index ( quintile)**						
1 (less deprived)	19.5	17.7	19.1	16.0	15.8	4.3
2	20.0	19.8	20.6	18.9	18.6	4.5
3	19.6	19.7	19.6	19.8	19.9	4.8
4	19.5	19.9	19.3	20.6	20.1	4.8
5 (most deprived)	20.1	21.8	20.3	23.6	24.4	5.4
* Missing*	1.1	1.1	1.1	1.1	1.1	4.8
**Municipality of residence**						
Urban	77.5	78.7	79.3	78.3	80.6	4.9
Rural	21.8	20.7	20.1	21.1	18.8	4.4
* Missing*	0.6	0.6	0.6	0.6	0.6	4.8
**Emergency department in the municipality of residence**	29.5	31.1	30.4	32.3	35.2	5.4
* Missing*	1.0	0.9	0.9	0.9	0.9	4.8
**At least one LTD**	4.0	12.0	13.4	13.4	33.9	13.5
Boys	4.6	12.0	13.2	13.6	33.5	13.4
Girls	3.3	11.9	13.6	13.3	34.3	13.8
**10 most frequent LTDs (ICD-10) for each outcome**:						
- Pervasive developmental disorders	0.53	0.93	1.15	0.73	1.07	5.5
- Asthma	0.24	0.58	0.63	0.63	1.33	11.0
- Specific developmental disorders of speech and language	0.17	0.24	0.28	0.20	0.22	4.4
- Epilepsy	0.17	0.67	0.70	0.87	2.16	15.5
- Unspecified mental retardation	0.16	0.42	0.52	0.38	0.73	8.3
- Type 1 diabetes mellitus	0.15	0.89	0.78	1.19	1.31	7.1
- Scoliosis	0.15	0.31	0.33	0.32	0.52	8.0
- Specific developmental disorders of scholastic skills	0.12	0.15	0.17	0.14	0.17	5.4
- Mixed disorders of conduct and emotions	0.10	0.15	0.14	0.17	0.27	8.6
- Mixed specific developmental disorders	0.09	0.14	0.16	0.14	0.18	6.2
- Congenital malformations of cardiac septa	0.08	0.25		0.33	0.87	16.7
- Cerebral palsy	0.07	0.31	0.39			0.0
- Other specified congenital malformation syndromes affecting multiple systems	0.06	0.28	0.31	0.33	0.79	13.5
- Sickle-cell diseases	0.04	0.37	0.46	0.51	2.17	28.1
- Disorders related to short gestation and low birth weight. not elsewhere classified	0.04				0.88	
- Lymphoid leukaemia	0.02				1.30	
- Cystic fibrosis	0.02		0.38			
- Malignant neoplasm of brain	0.02				0.81	

SSH: short stay hospital

IQR: interquartile range

LTD: long-term disease status

ICD-10: International Classification of Diseases 10th Revision

At least one LTD was noted for 4.0% of children (Boys 4.6%, girls 3.3%) for 13% of hospitalised and 34% readmitted children. Six of the 10 most frequent LTDs before 18 years of age were classified as “mental and behavioural disorders”, the most frequent being “Pervasive developmental disorders” (0.53%). Asthma (0.24%) was the most frequent somatic LTD, followed by epilepsy (0.17%). Regardless of the length of stay, the most frequent LTD for children who were hospitalised was pervasive developmental disorders (0.93%), followed by somatic diseases: type 1 diabetes mellitus (0.9%), epilepsy, or asthma (0.6% each). These LTDs were also the most frequent among children who had a SSH < 1 night, along with sickle-cell disease (0.5%), cerebral palsy, and cystic fibrosis (0.4% each). The most frequent LTDs for children who had a 30-day readmission ≥ 1 night were sickle-cell disease and epilepsy (2.2% each) followed by asthma, type 1 diabetes, and lymphoid leukaemia (1.3% each).

### Factors associated with SSH admission or readmission

The results for models adjusted for all variables showed children < 1 year of age to have the highest RR for SSH ≥ 1 (10.4) and readmission (14.8) relative to 5 to 9 year olds, followed by one-year-olds (Table 2, Additional file). Then, following a decrease with age, there was a slight increase for adolescents (14 to 17 years), mainly for SSH < 1 night (RR = 1.9). Girls were less often admitted than boys for each outcome and similar for readmission. Children with CMUC had a higher risk of being admitted, mainly for a SSH ≥ 1 night (RR = 1.4) and readmission (1.6). Children living in a rural municipality had a low risk of being admitted for a SSH < 1 night (RR = 0.9) but, conversely, a higher risk for a SSH ≥ 1 night (RR = 1.08). The presence of an ED in the municipality of residence was essentially associated with a higher risk of being readmitted (RR = 1.07).

**Table 2:** Adjusted sociodemographic characteristics and long-term disease associated with at least one short stay hospitalisation type for subjects < 18 years of age in 2018 and followed one year after their birth or birthday

	RR adjusted: full model (95% CI)				
	SSH stay	SSH stay < 1 night	SSH stay ≥ 1 night	SSH readmission ≥ 1 night	
**Age** (Years)					
< 1	4.79 (4.76–4.82)	1.12 (1.11–1.13)	10.43 (10.35–10.52)	14.93 (14.55–15.32)	
1	2.25 (2.24–2.27)	1.74 (1.72–1.75)	3.17 (3.14–3.20)	3.95 (3.82–4.09)	
2–4	1.71 (1.70–1.71)	1.70 (1.68–1.71)	1.74 (1.73–1.76)	1.84 (1.79–1.90)	
5–9	1	1	1	1	
10–13	0.87 (0.86–0.87)	0.80 (0.80–0.81)	0.97 (0.96–0.98)	1.08 (1.05–1.11)	
14–17	1.77 (1.76–1.78)	1.95 (1.94–1.97)	1.47 (1.45–1.48)	1.70 (1.65–1.74)	
**Girls**	0.84 (0.83–0.84)	0.76 (0.76–0.76)	0.93 (0.92–0.93)	0.97 (0.96–0.99)	
**Complementary universal health insurance**	1.20 (1.20–1.21)	1.06 (1.05–1.06)	1.39 (1.39–1.40)	1.61 (1.58–1.64)	
**Residence in a rural municipality**	0.99 (0.98–0.99)	0.91 (0.90–0.92)	1.08 (1.07–1.09)	1.00 (0.98–1.03)	
**Emergency department in the municipality**	1.00 (0.99-1.00)	0.99 (0.98–0.99)	1.02 (1.01–1.03)	1.07 (1.05–1.09)	
**LTD**					
• Pervasive developmental disorders	1.67 (1.64–1.70)	1.79 (1.76–1.84)	1.49 (1.45–1.53)	1.67 (1.54–1.80)	
• Asthma	1.99 (1.94–2.03)	1.93 (1.87–1.99)	2.27 (2.20–2.34)	3.21 (3.00-3.44)	
• Specific developmental disorders of speech and language	1.30 (1.25–1.34)	1.34 (1.28–1.40)	1.21 (1.15–1.28)	0.98 (0.83–1.17)	
• Epilepsy	2.86 (2.80–2.93)	2.60 (2.53–2.68)	4.02 (3.91–4.13)	5.88 (5.56–6.22)	
• Unspecified mental retardation	1.86 (1.81–1.91)	2.02 (1.95–2.09)	1.73 (1.66–1.80)	1.86 (1.70–2.05)	
• Type 1 diabetes mellitus	5.91 (5.80–6.02)	4.27 (4.16–4.39)	9.61 (9.39–9.83)	8.44 (7.87–9.05)	
• Scoliosis	1.85 (1.80–1.91)	1.69 (1.62–1.76)	2.35 (2.25–2.46)	2.60 (2.33–2.91)	
• Specific developmental disorders of scholastic skills	1.19 (1.14–1.25)	1.16 (1.10–1.23)	1.22 (1.14–1.30)	1.05 (0.86–1.27)	
• Mixed disorders of conduct and emotions	1.35 (1.29–1.42)	1.06 (0.99–1.13)	1.78 (1.68–1.89)	2.01 (1.73–2.33)	
• Mixed specific developmental disease	1.46 (1.39–1.52)	1.42 (1.33–1.50)	1.50 (1.40–1.61)	1.43 (1.19–1.72)	
• Sickle-cell disease	7.41 (7.21–7.63)	9.56 (9.24–9.90)	9.50 (9.18–9.84)	34.94 (33.10-36.88)	
• Cerebral palsy	3.14 (3.04–3.24)	3.53 (3.40–3.67)			
• Other specified congenital malformation syndromes affecting multiple systems	3.11 (3.01–3.22)	3.34 (3.20–3.48)	3.36 (3.22–3.51)	4.68 (4.28–5.12)	
• Congenital malformations of cardiac septa	1.78 (1.72–1.84)		2.10 (2.01–2.19)	2.79 (2.56–3.04)	
• Disorders related to short gestation and low birth weight				4.49 (4.13–4.89)	
• Lymphoid leukaemia				60.21 (56.16–64.55)	
• Malignant neoplasm of the brain				31.22 (28.57–34.11)	
• Cystic fibrosis		16.03 (15.43–16.66)			
• Other LTD (At least one)	3.40 (3.38–3.42)	3.43 (3.40–3.46)	4.24 (4.21–4.28)	12.14 (11.89–12.38)	

SSH: short stay hospital

RR Relative risk

LTD: long term disease status

95% CI: 95% Confidence Interval

The increased risk according to type of LTD differed depending on the outcome. The most elevated RR for a SSH ≥ 1 night was found for sickle-cell disease (RR = 9.5) and type 1 diabetes mellitus (RR = 9.6). The highest RR for a SSH < 1 night was found for cystic fibrosis (RR: 16) and sickle cell disease (RR = 9.6). The LTDs that led to an increased risk of 30-day readmission ≥ 1 night were lymphoid leukaemia (RR: 60.2), malignant neoplasm of the brain (RR: 31.2), and sickle cell disease (RR: 34.9).

### Primary and other healthcare use

At least one annual GP or paediatrician ambulatory visit was found for 88% of all children included (median number: 3, IQR = 2–6), paediatrician only for 17%, and other specialist for 39.5% (Table 3). Moreover, children also had outpatient hospital consultations (GP or paediatrician 8.5%). At least one annual ED visit, with or without SSH admission, was found for 24% (median number: 1, IQR = 1–2), psychiatric hospital admission for 0.31%, rehab admission for 0.23%, and hospitalisation at home for 0.04%. The frequency was higher for visits to least one other specialist than a paediatrician or GP relative to the other groups for children with a SSH < 1 night. Children who were admitted for a SSH ≥ 1 night or 30-day readmission had more frequent visits to an ambulatory paediatrician, nurse, physiotherapist, or outpatient consultation during the year. In addition, children readmitted for hospitalisation also had the highest rate of ED visits (87%), as well as those admitted to a psychiatric hospital (3.2%), rehabilitation facility (3.6%), or home hospitalisation (2.7%).

**Table 3:** One year of healthcare service use of subjects < 18 years of age in 2018 and followed one year after their birth or birthday with at least one short stay hospitalisation according to length or 30-day readmission

	At least one				
	Total	SSH stay	SSH stay < 1 night	SSH stay ≥ 1 night	SSH readmission ≥ 1 night
N children	13.211.235	1.157.701	650.043	588.631	55.509
**Ambulatory consultations**					
GP or paediatrician (%)*	88.0	92.9	91.6	94.4	94.0
Median [IQR]	3 [2–6]	5 [3–9]	4 [2–7]	6 [3–11]	7 [4–12]
GP (%)*	83.6	88.3	87.6	89.2	88.6
Median [IQR]	3 [2–5]	4 [2–7]	4 [2–6]	5 [2–8]	5 [3–9]
Paediatrician (%)*	17.3	25.2	20.7	30.6	33.6
Median [IQR]	2 [1–4]	3 [1–7]	2 [1–5]	4 [2–9]	5 [2–9]
Other specialists (%)*	39.5	53.9	66.0	40.2	41.2
Median [IQR]	1 [1–2]	2 [1–3]	2 [1–3]	2 [1–3]	2 [1–3]
Nurse (%)*	8.1	23.0	25.8	21.9	31.7
Median [IQR]	1 [1–2]	2 [1–8]	2 [1–8]	2 [1–9]	4 [1–16]
Physiotherapist (%)*	7.1	16.7	13.9	21.2	29.7
Median [IQR]	8 [4–15]	9 [4–20]	11 [5–26]	8 [4–19]	11 [5–27]
**Outpatient consultation**					
GP or paediatrician (%)*	8.5	19.5	18.2	23.2	39.8
GP (%)*	5.6	8.5	9.3	8.1	10.7
Paediatrician (%)*	3.1	11.9	9.8	16.3	32.5
**Emergency department**					
ED visit (%)*	23.6	57.8	42.2	78.2	86.9
Median [IQR]	1 [1–2]	1 [1–2]	1 [1–2]	2 [1–3]	3 [2–4]
**Hospitalisation**					
Psychiatric hospital (%)*	0.31	1.08	0.71	1.60	3.19
Rehabilitation (%)*	0.23	1.10	1.08	1.47	3.59
At home (%)*	0.04	0.44	0.25	0.81	2.70

SSH: short stay hospital; GP: general practitioner; and IQR: interquartile range

*At least one

### Diagnosis by SSH admission type

Among the 1.628 million SSH stays, the most frequent chapter of principal diagnoses (Table 4) was diseases of the digestive system (14.8%), followed by diseases of the respiratory system (13.9%), injury, poisoning and other consequences of external causes (8.8%), diseases of the genitourinary system (7.5%), symptoms signs and abnormal clinical and laboratory findings, not elsewhere classified (7.3%), and neoplasms (5.3%). The detailed diagnoses consisted of embedded and impacted teeth (7%), chronic diseases of the tonsils and adenoids (4.4%), redundant prepuce, phimosis, and paraphimosis (3.9%), acute bronchiolitis (2.7%), non-suppurative otitis media (2.4%), and asthma (2.1%).

**Table 4:** Diagnoses of one-year short stay hospitalisations and 30-day readmissions of subjects < 18 years-old in 2018 and 10 most frequent ICD 10 diagnoses according to length of stay or 30-day readmission

	At least one			
Diagnoses	SSH stay	SSH stay < 1 night	SSH stay ≥ 1 night	SSH readmission ≥ 1 night
N stay	1,628,594	858,018	770,576	86,170
	%	%	%	%
**ICD-10 Chapter**				
Certain infectious and parasitic diseases	4.68	1.18	8.57	7.46
Neoplasms	5.29	8.14	2.11	11.91
Diseases of the blood and blood-forming organs	2.53	3.13	1.85	5.93
Endocrine, nutritional and metabolic diseases	3.61	4.73	2.36	2.98
Mental and behavioural disorders	2.78	2.54	3.05	4.19
Diseases of the nervous system	2.80	2.21	3.45	4.89
Diseases of the eye and adnexa	1.04	1.55	0.46	0.41
Diseases of the ear and mastoid process	3.36	5.29	1.21	0.76
Diseases of the circulatory system	0.91	0.53	1.33	1.29
Diseases of the respiratory system	13.86	9.04	19.22	15.69
Diseases of the digestive system	14.83	21.40	7.52	7.80
Diseases of the skin and subcutaneous tissue	2.35	2.94	1.69	1.42
Diseases of the musculoskeletal system and connective tissue	2.92	2.93	2.91	2.60
Diseases of the genitourinary system	7.53	10.45	4.28	4.54
Pregnancy, childbirth and the puerperium	0.46	0.47	0.44	0.65
Certain conditions originating in the perinatal period	5.27	0.30	10.81	3.03
Congenital malformations, deformations and chromosomal abnormalities	4.56	5.35	3.68	3.26
Symptoms, signs and abnormal clinical and laboratory findings,	7.30	4.45	10.46	12.09
Injury, poisoning and certain other consequences of external causes	8.81	6.50	11.37	5.46
Factors influencing health status and contact with health services	5.13	6.87	3.20	3.63
**10 most frequent ICD-10 diagnosis for each outcome**				
Embedded and impacted teeth (K01)	7.04	13.19		
Chronic diseases of tonsils and adenoids (J35)	4.45	5.62	3.16	
Redundant prepuce, phimosis and paraphimosis (N47)	3.93	7.37		
Acute bronchiolitis (J21)	2.68		5.32	5.46
Non suppurative otitis media (H65)	2.41	4.26		
Asthma (J45)	2.14	1.28	3.10	2.11
Other gastroenteritis and colitis of infectious and unspecified origin (A09)	2.04		3.51	2.58
Intracranial injury (S06)	1.50		2.05	
Lymphoid leukaemia (C91)	1.41	2.30		2.83
Disorders related to short gestation and low birth weight (P07)	1.34		2.80	
Other orthopaedic follow-up care (Z47)		1.91		
Dentofacial anomalies [including malocclusion] (K07)		1.53		
Dental caries (K02)		1.35		
Chronic kidney disease (N18)		1.31		
Acute appendicitis (K35)			2.65	
Viral and other specified intestinal infections (A08)			2.63	2.57
Acute tubulo-interstitial nephritis (N10)			2.03	1.77
Respiratory distress of newborns (P22)			1.76	
Epilepsy (G40)				2.22
Other aplastic anaemias (D61)				2.12
Fever of other and unknown origin (R50)				1.89
Sickle-cell disease (D57)				1.86

SSH: short stay hospital

ICD-10: International Classification of Diseases 10th Revision

The diagnosis for stays < 1 night (858,018, 58% of admissions) more often concerned the digestive system Chap. (21%), including embedded and impacted teeth (13%), as well as diseases of the genitourinary system (10%), redundant prepuce, phimosis, and paraphimosis (7%), respiratory diseases (9%), and neoplasms (8%), particularly lymphoid leukaemia (2%). Stays ≥ 1 night (770,576, 47% of admissions) more often concerned a diagnosis in the chapter of respiratory diseases (19%), including acute bronchiolitis (5%), chronic diseases of the tonsils and adenoids (3%), and asthma (3%), as well as injury, poisoning, and certain other consequences of external causes (11%), including intracranial injury (2%), and conditions originating in the perinatal period (11%), including disorders related to short gestation and low birth weight (3%) and respiratory distress of the new-born (2%).

The most frequent diagnoses for 30-day readmission ≥ 1 night (86,170, 5.3%) were in the chapter of signs and abnormal clinical and laboratory findings, not elsewhere classified (12%), neoplasms (12%), with 3% of readmission with a diagnosis of lymphoid leukaemia, diseases of the blood (6%), including other aplastic anaemias (2%) and sickle-cell disease (2%), mental diseases (4.2%), and diseases of the nervous system (4.9%), particularly epilepsy (2%). Diagnosis included in the chapter “Symptoms, signs and abnormal clinical and laboratory findings” were more frequent for SSH readmission (12.1%) and SSH > 1 night (10.5%). Conversely, diagnosis included in the chapter Factors influencing health status and contact with health services were slightly higher for SSH < 1 night (6.9%). Globally, both chapters totalized 12.4% of stays.

### Diagnosis of potentially preventable SSH

Among hospitalisations of children between 0 and 10 years of age, potentially preventable hospitalisation represented 25.8% of all SSH stays, 33.9% of SSH stays ≥ 1 night, and 30.9% of readmissions (Table 5). The most frequent diagnoses were dehydration and gastroenteritis (6.2% of all stays, 9.93% of stays ≥ 1 night), acute bronchiolitis (4.2% of all stays, 7.4% of stays ≥ 1 night), ear, nose, and throat infections (4.0% of all stays, 7.6% of stays < 1 night), and asthma (3.3% of all stays).

**Table 5:** Potentially preventable hospitalisations of children between the age of 0 and 10 (included)

Diagnoses potentially preventable hospitalisation (ICD-10)	SSH stay	SSH stay < 1 night	SSH stay ≥ 1 night	SSH readmission ≥ 1 night
Total admission (N)	1,035,883	482,612	553,271	59,160
	%	%	%	%
Acute bronchiolitis	4.21	0.55	7.41	7.95
Acute upper respiratory tract infection excluding croup	1.80	0.53	2.90	2.52
Asthma	3.29	1.56	4.81	3.54
Bronchiectasis	0.01	0.01	0.01	0.01
Bacterial meningitis	0.04	0.00	0.08	0.08
Constipation	0.24	0.12	0.35	0.34
Convulsions and epilepsy, including eclampsia	0.78	0.32	1.18	0.58
Croup, acute laryngitis, tracheitis	0.52	0.52	0.52	0.32
Dehydration and gastroenteritis	6.23	1.98	9.93	9.09
Dental conditions	1.12	2.23	0.15	0.08
Dermatitis/eczema	0.08	0.02	0.13	0.13
Ear, nose and throat infections	4.00	7.56	0.89	0.65
Gastro-oesophageal reflux	0.50	0.22	0.74	1.24
Influenza and pneumonia (including viral and bacterial /unspecified pneumonia	1.20	0.15	2.11	1.74
Meningitis (viral/other/unspecified)	0.23	0.02	0.41	0.22
Meningococcal disease	0.01	0.00	0.02	0.01
Nutritional deficiencies	0.13	0.05	0.21	0.42
Other vaccine preventable diseases	0.08	0.01	0.15	0.18
Osteomyelitis	0.12	0.02	0.21	0.22
Rheumatic heart disease, including acute rheumatic fever	0.00	0.00	0.00	0.01
Skin infections	0.74	0.56	0.90	0.86
Tuberculosis	0.02	0.00	0.03	0.05
Urinary tract infection, including pyelonephritis (children aged ≥ 5 years)	0.29	0.10	0.45	0.37
Viral infection of unspecified site	0.20	0.06	0.32	0.29
Total	25.8	16.6	33.9	30.9

SSH: short stay hospital

ICD-10: International Classification of Diseases 10th Revision

*Anderson P, Craig E, Jackson G, et al. Developing a tool to monitor potentially avoidable and ambulatory care sensitive hospitalisations in New Zealand children. N Z Med J 2012;125:25–37

## Discussion

This first French quasi-exhaustive one-year study on 13.2 million children < 18 years of age (94.4% of the population) provides rare data on the type of SSH admission according to their characteristics: 8.8% had at least one SSH admission, (4.9% < 1 night, 4.4% ≥ 1 night) and 4.8% a 30-day readmission ≥ 1 night. Factors associated with more frequent SSH ≥ 1 night or readmission were mainly an age < 2 years, being male, having CMUC (household income under the threshold of poverty), social deprivation, residence in a rural area for SSH ≥ 1 night and the presence of an ED in the municipality for readmission. SSH admission < 1 night was moderately more frequent for infants between 1 and 4 years of age, males, and those living in a deprived municipality and much more frequent for those with at least one LTD. Admitted children had more frequent primary care visits during the year. This was particularly true for those who were readmitted. This was also true for those with at least one LTD overall or a specifically detailed LTD, depending on the outcome considered. Overall, 25.8% of SSHs of children < 10 years of age were potentially preventable.

### SSH admission

Between 2009 and 2012, a wide range of standardized hospital admission rates per 100 child years (0 to 19 years) were reported for seven European countries: between 9.4 for Spain and 19.6 for Germany, with France in sixth place (13.5). Among all causes, a varying percentage of potentially avoidable SSHs was suggested [1]. In the USA, a slight increase in the number of discharges was observed for all SSH stays between 2000 and 2007 (excluding mother’s pregnancy and delivery), with rates of discharge of for 2007 of 6% for 1 to 4 year olds, 1.7% for 5 to 9 year olds, 1.0% for 10 to 14 year olds, and 1.8% for 15 to 17 year olds and an overall rate of 3%, lower than our study in France with universal medical coverage [4]. From 2010 to 2016, the total number of index admissions decreased by 21.3% in a representative US sample (children < 1 year excluded), but the percentage of admissions for children with complex chronic conditions increased by 5.7% (7% in 2016) [5]. This last point is explained by the increase in the prevalence of these children following progress in healthcare and survival. In 2018, the top five principal diagnosis for SSH for children from 1 to 17 years of age in the USA were acute bronchitis (0.13%), depressive disorders (0.12%), asthma (0.1%), pneumonia (0.09%), and epilepsy (0.08%) [27]. In our study, respiratory diseases was the most frequent diagnosis for SSH ≥ 1 night and digestive diseases (including dental care) for SSH < 1 night: dental care was most often performed during one-day admission, as for certain other genitourinary and ENT diseases.

There is an abundance of literature on what are doing to reduce the volume, duration and severity of hospital stays for both children and adults. In France, the main action point on hospital organisation is characterised by a dramatic increase in the number of recommendations and policies to develop ambulatory care, including one-day stays for surgery and ambulatory surgery and anaesthesia, as in many countries. Children are generally in sufficiently good health to undergo simple surgical procedures and diagnostic or therapeutic acts requiring short general anaesthesia [28, 29] Individualizing children with stays < 1 night reduces the child SSH rate from 8.8 to 4.4% (comprising 53% of children and stays in SSH), but with a smaller impact on the days spent in hospital. These children were the youngest relative to the others, with a peak of SSHs between 1 and 4 years of age and were more often boys, slightly more deprived and living less in a rural area, and with various somatic or psychiatric LTDs. They more frequently visited ambulatory specialists other than paediatricians. The most frequent diagnoses were related to surgical procedures, such as for teeth, tonsils, and adenoids, prepuce, ears/mastoid, and congenital malformations. Nevertheless optimisation could be improved by the increase of primary care density in some regions, mainly specialists [30].

Concerning various type of stays some may be more often explored and detailed. This is the case of specifically isolate units stays of varying lengths for clinical decision or / observation stays in USA. Nevertheless, in the SNDS there is no specific information on this type of unit. Some proxies may be detailed asthe highest adjusted RR for SSH stay≥ 1 night for children living in a rural municipality. This point could be explored and detailed suing the main diagnosis included in the chapters “Symptoms, signs and abnormal clinical and laboratory findings” and “Factors influencing health status and contact with health services and totalizing 12.4% of stays.

Proportion of preventable SSHs which has been infrequently analyzed among children and commented must be detailed in France. In Italy, 10.5% of hospitalisations were judged to be preventable in 2016. Dehydration (30%), pneumonia (18%), seizures (16%), and bronchitis (3%) accounted for 90% of preventable paediatric hospitalisations. They are mostly related to acute illness, highlighting difficulties in managing acute diseases of the youngest in primary care [31]. Nevertheless, the criteria for preventable admissions and their numbers or coding (ICD9 vs. ICD10) varied between studies, as did the age range: 50% of potentially preventable hospital charges were associated with asthma and bacterial infection in the 2006 kid’s inpatient database [32]. Furthermore 15% of hospital discharges followed preventable hospitalisation for children < 21 years of age with a non-complex chronic disease and 5% for children with a medically complex condition [33]. In comparison to a New-Zealand study, which found 34.8% of potentially preventable hospitalisation using their definition, we found a lower rate (25.8%) for all SSH. However, the rates were closer if only SSH ≥ 1 night was considered (33.9%) or readmissions ≥ 1 night (30.9%). Neither definition defines a potentially preventable hospitalisation in practice, nor what proportions of potentially preventable hospitalisation admissions are realistically preventable. Indeed, the authors of the definition for children noted that not all potentially preventable hospitalisation admissions may have been prevented at the individual level nor through primary healthcare, acknowledging the role of wider factors, such as socioeconomic status, housing, and government policies [25, 26].

Proportion of 30-day readmission rate ≥ 1 night (in the same hospital or not) must be also deeply explored. Among the leading causes of readmission In the USA (2013-14), more than 50% of 30-day readmissions after acute conditions occurred within 15 days after discharge, whereas readmissions after chronic conditions occurred more uniformly throughout the 30 days after discharge [9]. Most 30-day readmissions after chronic conditions were for the same diagnosis, or closely related conditions, as the index admission [28]. SSH readmission rates varied according to the diagnosed condition, length of stay, and hospital [15]. In the USA, the 30-day readmission rate of unplanned readmission for children aged 1 to 17 years was essentially stable between 2009 (5.5%) and 2014 (5.9%). In 2014, the rates ranged from 2.6% after hospitalizations for appendectomy to 19.1% after hospitalizations for sickle cell disease [7]. In our study, we found frequent readmission for children with LTDs, chronic by definition, and sickle cell disease, type 1 diabetes, asthma, cystic fibrosis, or epilepsy as the principal diagnosis. Other studies found similar rates for a one-year follow-up (5–8%), depending on the year of the study [5, 9, 14, 15, 17]. A cross sectional study based on interviews found that 30% of 30-day readmissions are preventable and could be avoided [33].

Thus, various actions can be developed with general practitioners or specialists impacting readmission and preventable admission rates. However they must take account of the more or less insufficient supply of care in area of residence frequently deprived [30].

The reasons reported for unplanned SSH and readmissions are organizational, In particular out-of-hospital primary care (transition between primary care and hospital, continuity of scheduled care), the adequacy of treatment, disease progression, and the recurrence and complications of pathologies and treatments, as well as specific concerns for children with medically complex conditions [12, 34–37]. Individual characteristics have also been found to be associated with unplanned SSH and readmission: lower age, being male, insurance coverage, ethnicity, low income, and living in a social deprivation area, with or without an ED in the municipality, in a rural zone, mostly as in our study [13, 14, 38–44]. Girls were less often admitted than boys, in particular, for hospitalizations < 1 night, or readmitted. This may be due to variations or the inexistence of specific one-day surgeries. Rurality increased SSHs ≥ 1 night, conversely to those of < 1 night. This can be explained, at least in part, by the need for longer monitoring before the return to home, as well as the greater likelihood of being at a socioeconomic disadvantage or having a complex chronic disease [39].

The most common factors reported for hospital readmission are comorbidities, the length of stay, severity of the illness, and principal procedures. A comorbidity can be ascribed to long-term chronic or medically complex conditions. A definition of medical complexity has been proposed [17]: “children with a chronic physical, developmental, behavioural or emotional condition requiring health and related services of a type or amount beyond that required by children generally, defined for the general population or in-hospital patient criteria”, close to that used for LTDs “chronic diseases of higher severity that require costly, regular, and long-term care and are potentially life-threatening or lead to disabilities”. A recent study with differences in coding criteria for medical complexity reported a prevalence between 0.67% and 11.4% [18]. Here, we found a prevalence of LTDs of 4% (0–17 years) that increased with age. Higher use of healthcare services has also been found by studies on children with complex chronic conditions relative to children without such conditions: a higher annual proportion of primary ED visits and hospital admissions and readmissions [8, 18].

Two complementary descriptive studies on the same population detailed certain characteristics, risk factors, and their association. The first reported that the rate of children with LTDs, such as psychiatric LTDs, increased with age, and that they had more ambulatory specialist visits, ED visits, and SSH and psychiatric hospital admissions [30]. The second study reported that LTDs were more frequent for children living in low-income households and municipalities classified in the most deprived index quintiles [45]. Moreover, they were less likely to have seen a paediatrician, another specialist, or dentist. Indeed, the density of specialists and paediatricians has been decreasing in the municipalities ranked in the highest deprivation quintiles. 50% of children living in a low-income household and with a higher frequency of LTDs often live in a municipality with an ED, which may facilitate their use [30].

### Strengths and limitations

The main strength of this study was the use of the SNDS, which allowed us to include more than 13 million children, representing 94.4% of the metropolitan French population in this age group listed by the National Institute for Statistics and Economic Studies, and which exhaustively collects hospital discharge database information.

Nevertheless, we observed a difference with the National Institute for Statistics and Economic Studies population, which increased with age, possibly due to the non-inclusion of children who had no reimbursements during the year in our study, leading to a slight overestimation of healthcare consumption. Most individuals without reimbursements during the year appeared to be adolescents. In addition, younger children born alive but not discharged from the hospital may have not been included in the study because they did not have an outpatient refund. In addition, their CMUC status could not be determined. The social deprivation index we used in this study is an indicator for the level of the smallest administrative unit. It does not necessarily indicate the social disadvantage of each individual living in the municipality and was not completely independent of the CMUC status at the individual level, which was also more frequent in communities ranked in the more deprived quintiles.

It is not easy to differentiate planned and unplanned admissions in the SNDS. Generally, SNDS users select those admitted via ED visits followed by an intensity care unit stay, which is not the case for all unplanned admissions, such as a diagnosis of trauma without intensive care. In this study, 78% of children had an ED visit for a hospital stay ≥ 1 night and 87% for readmission ≥ 1 night. Nevertheless, some may have also been directly admitted to intensive care. For stays < 1 night, most admissions should be planned for ambulatory activity but 42% also followed ED visits.

LTDs require regular, costly, long-term care, and may be potentially life-threatening or disabling. Moreover, specific to France, the LTD status guarantees 100% reimbursement and is defined on a stable national list by decree. Indeed, certain patients may not yet have been diagnosed or may have had low-intensity symptoms, with little or no use of healthcare services at the onset of their disease, and may have been eligible for access to free healthcare without the need for LTD registration or had a primary LTD related to another LTD. Nevertheless, this underestimation is unlikely to concern patients with the most severe conditions or disease evolution, who have greater healthcare needs. Analyses of LTDs focus specifically on a stable national list rather than algorithms for chronic diseases. Finally, parents may refuse to apply for a LTD for their children, for example, for psychiatric diseases.

## Conclusion

Our results for this and complementary studies offer insightful baseline information on variations in healthcare use by children. These data must also be considered with those of specific studies, such as at the regional level, to adapt policies and future research according to multiple characteristics, as well as plan and adapt care to the needs and access requirements of patients at different levels.

### Electronic supplementary material

Below is the link to the electronic supplementary material.

Supplementary Material 1

## Data Availability

All SNDS data are anonymous and individually linkable. Access to data is subject to prior training and authorisation and needs approval by the independent French data protection authority (“Commission Nationale Informatique et Libertés”). Data cannot be shared publicly because it is forbidden by law (sensitive individual data). Data are available from the Health Data Hub (contact via hdh@health-datahub.fr) for researchers who meet the criteria for access to confidential data.

## Abbreviations

CMUC: Complementary health universal coverage
ED: Emergency Department
ICD-10: International Classification of Diseases 10th revision
LTD: Long-Term chronic Disease
SNDS: French national health data system
SSH: Short-Stay Hospitalisation
IQR: Interquartile range
RR: Relative risk
95%CI: 95% confidence interval
