# Supplementary material for: Factors associated with hospital admission and 30-day readmission for children less than 18 years of age in 2018 in France: a one-year nationwide observational study
Source: BMC Health Serv Res. 2023 Aug 23;23:901. doi: 10.1186/s12913-023-09861-2 (PMC10464416; doi:10.1186/s12913-023-09861-2)
Supplement: Supplementary file 1 — Supplementary Material 1 [file 12913_2023_9861_MOESM1_ESM.docx]

**Additional file 1**

Sociodemographic characteristics and long-term disease associated with at least short stay hospitalisation type for subjects < 18 years of age in 2018 and followed one year after their birth or birthday, adjusted for age and sex

|  | **Crude RR (95% CI)** | | | |
| --- | --- | --- | --- | --- |
|  | **SSH stay** | **SSH stay**  **< 1 night** | **SSH stay**  **≥ 1 night** | **SSH readmission**  **≥ 1 night** |
| **Age (Years)** |  |  |  |  |
| < 1 | 4.59 (4.56-4.61) | 1.08 (1.06-1.09) | 9.84 (9.76-9.91) | 12.60 (12.27-12.95) |
| 1 | 2.19 (2.17-2.20) | 1.68 (1.67-1.70) | 3.05 (3.02-3.08) | 3.52 (3.40-3.65) |
| 2-4 | 1.68 (1.67-1.69) | 1.67 (1.65-1.68) | 1.70 (1.69-1.72) | 1.73 (1.68-1.79) |
| 5-9 | 1 | 1 | 1 | 1 |
| 10-13 | 0.88 (0.88-0.89) | 0.82 (0.81-0.82) | 0.99 (0.98-1.00) | 1.13 (1.10-1.17) |
| 14-17 | 1.82 (1.81-1.83) | 2.01 (1.99-2.02) | 1.52 (1.51-1.54) | 1.85 (1.80-1.90) |
| **Girls** | 0.82 (0.82-0.83) | 0.75 (0.74-0.75) | 0.91 (0.90-0.91) | 0.91 (0.90-0.93) |
| **Complementary universal health insurance** | 1.28 (1.27-1.28) | 1.11 (1.11-1.12) | 1.52 (1.51-1.52) | 1.94 (1.91-1.98) |
| **Geographical social deprivation index** |  |  |  |  |
| 1 (less deprived quintile) | 1 | 1 | 1 | 1 |
| 2 | 1.09 (1.08-1.09) | 1.05 (1.04-1.05) | 1.15 (1.14-1.16) | 1.15 (1.11-1.18) |
| 3 | 1.10 (1.10-1.11) | 1.02 (1.01-1.03) | 1.22 (1.21-1.23) | 1.25 (1.21-1.28) |
| 4 | 1.12 (1.12-1.13) | 1.01 (1.00-1.02) | 1.28 (1.27-1.29) | 1.27 (1.24-1.31) |
| 5 (most deprived) | 1.19 (1.18-1.20) | 1.03 (1.02-1.03) | 1.43 (1.41-1.44) | 1.49 (1.45-1.53) |
| **Residence in a rural municipality** | 0.93 (0.93-0.94) | 0.90 (0.90-0.91) | 0.96 (0.95-0.96) | 0.83 (0.81-0.85) |
| **Emergency department in the municipality** | 1.08 (1.07-1.08) | 1.04 (1.03-1.05) | 1.14 (1.13-1.14) | 1.29 (1.27-1.32) |
| **At least one LTD** | 3.30 (3.28-3.31) | 3.73 (3.70-3.75) | 3.75 (3.73-3.78) | 12.39 (12.18-12.61) |
| **LTD** |  |  |  |  |
| - Pervasive developmental disorders | 1.75 (1.72-1.78) | 2.17 (2.13-2.22) | 1.38 (1.34-1.42) | 2.02 (1.86-2.19) |
| - Asthma | 2.46 (2.41-2.52) | 2.67 (2.59-2.75) | 2.68 (2.60-2.77) | 5.73 (5.33-6.16) |
| - Specific developmental disorders of speech and language | 1.37 (1.32-1.42) | 1.62 (1.55-1.70) | 1.14 (1.08-1.20) | 1.25 (1.05-1.50) |
| - Epilepsy | 4.06 (3.98-4.15) | 4.20 (4.08-4.32) | 5.27 (5.13-5.41) | 13.24 (12.50-14.01) |
| - Unspecified mental retardation | 2.62 (2.55-2.70) | 3.24 (3.14-3.35) | 2.37 (2.27-2.46) | 4.56 (4.14-5.03) |
| - Type 1 diabetes mellitus | 5.92 (5.81-6.03) | 5.18 (5.04-5.32) | 7.94 (7.76-8.13) | 8.75 (8.13-9.41) |
| - Scoliosis | 2.04 (1.98-2.11) | 2.21 (2.12-2.30) | 2.11 (2.02-2.21) | 3.42 (3.05-3.84) |
| - Developmental disorders of scholastic skills | 1.27 (1.21-1.32) | 1.42 (1.34-1.50) | 1.15 (1.08-1.23) | 1.42 (1.16-1.73) |
| - Mixed disorders of conduct and emotions | 1.52 (1.45-1.59) | 1.37 (1.29-1.46) | 1.74 (1.64-1.85) | 2.74 (2.34-3.22) |
| - Mixed specific developmental disorders | 1.63 (1.55-1.70) | 1.78 (1.67-1.89) | 1.54 (1.43-1.64) | 2.08 (1.72-2.53) |
| - Sickle-cell disease | 8.42 (8.19-8.67) | 10.43 (10.08-10.81) | 11.54 (11.14-11.95) | 49.72 (46.97-52.64) |
| - Cerebral palsy | 4.24 (4.11-4.37) | 5.38 (5.18-5.59) |  |  |
| - Other specified congenital malformation   syndromes affecting multiple systems | 4.63 (4.48-4.78) | 5.15 (4.93-5.37) | 5.35 (5.12-5.59) | 13.05 (11.88-14.33) |
| - Congenital malformations of cardiac septa | 3.04 (2.93-3.15) |  | 4.08 (3.90-4.26) | 10.72 (9.80-11.73) |
| - Disorders related to short gestation and low birth weight |  |  |  | 22.80 (20.87-24.92) |
| - Lymphoid leukaemia |  |  |  | 62.34 (57.94-67.08) |
| - Malignant neoplasm of the brain |  |  |  | 52.71 (48.05-57.83) |
| - Cystic fibrosis |  | 18.32 (17.62-19.04) |  |  |
| - Other LTD (At least one) | 3.65 (3.63-3.68) | 3.96 (3.93-3.99) | 4.23 (4.19-4.27) | 13.17 (12.91-13.43) |

RR Relative risk

95% CI: 95% Confidence Interval

SSH: short stay hospital

LTD: long term disease
